# Tobacco–barley rotation reshapes soil microbial communities and enriches culturable bacteria with plant growth-promoting potential

**DOI:** 10.3389/fpls.2026.1981771

**Published:** 2026-10-28

**Authors:** Zilin Li, Chengxiu Fu, Xun He, Zhufeng Shi, Yongqin Liao, Xufang He, Te Pu, Yanni Wang, Ming Ni, You Luo, Wei Wang, Jing Li, Xin He, Chenying Li, Xuewei Li, Peiwen Yang

**Affiliations:** 1 Yunnan Tobacco Company, Lijiang Company, Lijiang, China; 2 Institute of Agricultural Environment and Resources, Yunnan Academy of Agricultural Sciences, Kunming, China

**Keywords:** amplicon sequencing, barley, continuous-cropping obstacles, crop rotation, flue-cured tobacco, plant growth-promoting strains

## Abstract

**Introduction:**

Crop rotation is an important agronomic practice for alleviating the adverse effects of continuous tobacco cropping, yet the effects of preceding gramineous crops on the microbial communities and potential functions of tobacco-growing soils have not been systematically verified.

**Methods:**

Based on a three-year continuous field experiment, this study used a flue-cured tobacco–fallow system (CK) as the control and established a flue-cured tobacco–barley rotation system (DM). Soil chemical properties and enzyme activities, bacterial 16S rRNA and fungal ITS amplicon sequencing, culturable bacterial isolation, and greenhouse pot reinoculation experiments were integrated to evaluate changes in the soil environment and microbial communities following barley rotation and to verify the plant growth-promoting potential of candidate enriched isolates.

**Results:**

Compared with CK, DM significantly (*P* < 0.05) increased soil dissolved organic carbon, available phosphorus, available potassium, and hydrolyzable nitrogen by 19.65%, 3.23%, 5.45%, and 5.90%, respectively; sucrase, β-glucosidase, acid phosphatase, and N-acetyl-β-D-glucosaminidase activities increased by 23.80%, 66.28%, 15.23%, and 176.32%, respectively. Amplicon sequencing showed that barley rotation significantly altered soil bacterial and fungal community. *Sphingomonas* and *Burkholderia-Caballeronia-Paraburkholderia* were relatively enriched under DM and were significantly associated with multiple soil nutrient and enzyme activity variables; therefore, they were defined as candidate enriched bacterial taxa. Six culturable isolates belonging to these taxa were subsequently obtained from DM soil. *In vitro*, these isolates exhibited different combinations of functional phenotypes, including growth on nitrogen-free medium, phosphorus transformation, potassium solubilization, zinc solubilization, siderophore production, and organic-substrate degradation. Greenhouse pot experiments showed that mixed inoculation significantly promoted flue-cured tobacco growth and increased plant nitrogen, phosphorus, and potassium accumulation.

**Conclusion:**

Barley rotation was accompanied by significant changes in soil available nutrients, enzyme activities, and microbial community composition, whereas culturable isolates obtained from rotation-enriched taxa showed plant growth-promoting potential under controlled conditions. These findings support the possibility that rotation-enriched microorganisms participate in subsequent plant–soil nutrient feedbacks, although their direct mediating role in field rotation effects requires further verification. This study provides a theoretical basis and candidate microbial resources for understanding the microbial ecological effects of barley–tobacco rotation and screening potential plant growth-promoting bacteria.

## 1 Introduction

Flue-cured tobacco (*Nicotiana tabacum* L.) is an important cash crop in southwestern China (Liu M. et al., 2024). However, tobacco is highly sensitive to continuous cropping. Long-term continuous cropping can lead to the accumulation of allelochemicals such as phenolic compounds in the rhizosphere, disrupt plant membrane systems, photosynthesis, and related enzyme activities, and consequently inhibit plant growth and reduce tobacco yield and quality (Ma et al., 2023; Ren et al., 2026). Meanwhile, continuous cultivation can intensify soil nutrient depletion and quality degradation, promote the accumulation of potential pathogenic microorganisms, disturb microbial community structure, and aggravate soil-borne diseases (Chen et al., 2021; Wu H. et al., 2025). Previous studies have shown that long-term continuous cropping can markedly reduce flue-cured tobacco yield and is accompanied by decreases in soil available nitrogen, phosphorus, potassium, and organic matter (Liu et al., 2025; Wang K. et al., 2024). At the microbial level, continuous cropping is generally characterized by reduced bacterial diversity, declines in beneficial microorganisms, and enrichment of potential pathogenic fungi such as *Fusarium*, while also affecting microbial functions related to soil carbon, nitrogen, and other nutrient cycles (Gong et al., 2024; Li et al., 2024). Therefore, restoring rhizosphere microecological functions through appropriate cropping systems is an important scientific issue for alleviating continuous-cropping obstacles in tobacco and maintaining the productivity of tobacco-growing soils.

Crop rotation is an important agricultural practice for alleviating continuous-cropping obstacles and improving soil health. Previous studies have shown that different rotation systems can promote the growth of subsequent crops by improving soil physicochemical properties, increasing microbial diversity, enriching beneficial microorganisms, and regulating microbial interaction networks (Chen et al., 2018; Chen et al., 2020; Liu L. et al., 2024; Zong et al., 2024). However, soil legacy effects generated by different preceding crops are strongly species-specific. For example, legumes mainly increase soil nitrogen inputs through symbiotic nitrogen fixation by rhizobia (Peoples et al., 2009), whereas crops such as rapeseed and wheat can alter soil nutrient cycling and associated microbial taxa through differences in root exudates and residue quality (Kaloterakis et al., 2025). In tobacco rotation systems, previous studies have also demonstrated that preceding crops such as legumes and garlic can promote the growth of subsequent flue-cured tobacco by regulating soil structure, nutrient status, and microbial communities (Ren et al., 2024). Although these studies clearly demonstrate the benefits of crop rotation, most existing research has focused on outcome-oriented assessments of whether rotation improves soil properties and crop growth. The crop-specific microbial legacy effects formed by different preceding crops, particularly which core functional microbial groups actually participate in the growth and nutrient acquisition of subsequent flue-cured tobacco, remain insufficiently understood.

As a gramineous crop, barley (*Hordeum vulgare* L.) differs markedly from tobacco in taxonomy, root resource-use strategies, and pathogen host range. Barley roots can release readily available carbon sources such as sugars, while root activity during the growing season and residue return can alter the composition of soil organic substrates, thereby exerting selective pressure on rhizosphere microorganisms (Pacheco-Moreno et al., 2024; Robertson-Albertyn et al., 2017; Sun et al., 2025). In addition, phylogenetically distant rotation between Poaceae and Solanaceae may help interrupt the continuous proliferation of some host-preferential pathogens (Wei et al., 2026). Therefore, barley as a preceding crop may generate a soil microbial legacy distinct from that of legumes and other rotation crops through a process of “rhizosphere carbon input–environmental filtering–microbial community reassembly”.

Soil microbial legacy effects are considered an important mechanism mediating the influence of preceding crops on subsequent crop performance (Bakker et al., 2018; Jing et al., 2022). Preceding crops can filter soil microbial communities through root exudation, rhizosphere nutrient depletion, and residue inputs, allowing some microbial effects to persist after crop harvest and subsequently influence nutrient acquisition and growth of the following crop (Town et al., 2022; Wu J. et al., 2025). To date, several studies on tobacco rotation have reported that rotation can increase the relative abundance of beneficial bacteria, enhance bacterial diversity, and increase the complexity of microbial co-occurrence networks (Yan et al., 2024). However, an evidence chain linking community-level associations to strain-level functions is still lacking for the question of which rotation-enriched taxa possess verifiable plant growth-promoting functions. In the barley–tobacco system in particular, it remains necessary to determine which bacterial taxa are consistently enriched after barley harvest, whether these taxa contain culturable members with nutrient-transformation-related phenotypes, and whether representative strains can promote flue-cured tobacco growth and nutrient accumulation after reinoculation.

Accordingly, this study integrated a long-term field experiment, amplicon sequencing, culturable microorganism isolation, and pot reinoculation assays. We hypothesized that barley rotation would generate a soil microbial community distinct from that of the flue-cured tobacco–fallow system and enrich candidate bacterial taxa associated with nutrient transformation and plant growth promotion, and that some culturable representative strains from these taxa would have the potential to promote flue-cured tobacco growth and nutrient acquisition under controlled conditions. The objectives were to: characterize the responses of soil chemical properties, enzyme activities, and microbial communities after barley rotation; screen and isolate culturable representatives of candidate enriched bacterial taxa; and evaluate their plant growth-promoting potential through *in vitro* functional phenotyping and greenhouse pot experiments. This study aimed to establish a preliminary link between field-scale community changes and strain-level functional validation, thereby providing a theoretical basis and candidate microbial resources for screening potential plant growth-promoting bacteria.

## 2 Materials and methods

### 2.1 Site description

The field experiment was conducted in Luwa Village, Shigu Town, Yulong County, Lijiang City, Yunnan Province, with geographic coordinates of 26°50′ N, 99°54′ E. Situated in the Jinsha River valley, the experimental site has a dry-hot river-valley climate at an elevation of 1859 m (**Supplementary Figure S1**). During the experimental period, the local mean annual temperature was 21.4 °C, annual precipitation was 868.2 mm, and total sunshine duration reached 2421.4 h. The soil is classified as mountain sandy yellow soil. The baseline soil physicochemical properties prior to the experiment were as follows: pH 5.71, soil organic matter 46.23 g/kg, total nitrogen 2.42 g/kg, hydrolyzable nitrogen 237.69 mg/kg, total phosphorus 1.08 g/kg, available phosphorus 108.37 mg/kg, total potassium 19.77 g/kg, and available potassium 257.50 mg/kg. The flue-cured tobacco cultivar used in this study was ‘Yunyan 87’.

### 2.2 Experimental design

A field with a long history of flue-cured tobacco cultivation was selected, and a long-term fixed-site field experiment was initiated in late April 2022. Two cropping systems were established: a flue-cured tobacco–fallow system (CK) and a flue-cured tobacco–barley rotation system (DM). A randomized block design was used, with four independent field plots per treatment (eight plots in total). Each plot covered approximately 25.2 m^2^, buffer rows were established between plots, and each plot served as an independent experimental unit. During the flue-cured tobacco growing season, both treatments used the same tobacco cultivar, transplanting density, fertilization, irrigation, and pest and disease management. Both received distillers’ grains organic fertilizer (N + P_2_O_5_ + K_2_O = 6.2%) and compound fertilizer (N:P_2_O_5_:K_2_O = 15:15:15), with fertilizer type, application rate, and timing kept identical. The two cropping systems differed mainly during the non-tobacco season after tobacco harvest. In DM, barley was planted annually (row spacing approximately 0.10 m, plant spacing approximately 0.05 m, approximately 200 plants m^−2^), compound fertilizer was applied at 600 kg ha^−1^ during the barley season, and barley was harvested in early April of the following year. After harvest, 10–12 cm stubble was retained and the remaining residues were returned to the soil, with residue input of approximately 6,000 kg ha^−1^. In CK, no crop was planted during the corresponding period, and no barley-season fertilization or barley residue return was performed. Thus, this study compared the integrated effects of two complete cropping-management systems. Barley plant inputs, barley-season fertilization, and residue return were all components of the DM system and may have jointly influenced soil properties and microbial communities; therefore, the observed effects should not be interpreted as the effect of the barley plant alone.

### 2.3 Soil sample collection

Soil was collected in April 2025 after barley harvest and before flue-cured tobacco transplanting for the following season. CK and DM each comprised four independent field plots (n = 4), yielding eight independent soil samples in total. In each plot, topsoil (0–20 cm) was collected using a five-point sampling method. The five subsamples were thoroughly mixed to form one representative sample for that plot. The five-point sampling procedure was used only to reduce within-plot spatial heterogeneity and was not treated as independent biological replication; independent field plots were always the experimental units used for statistical analysis. Each composite soil sample was divided into three portions. The first portion was placed in a sterile container, refrigerated at 4 °C, and transported to the laboratory as soon as possible for isolation of culturable microorganisms. The second portion was used for microbial community DNA extraction and amplicon sequencing and was stored at −80 °C after cold-chain transportation. The third portion was placed in a clean resealable bag for determination of soil chemical properties and soil enzyme activities.

### 2.4 Determination of soil chemical properties and enzyme activities

Soil pH was measured by the potentiometric method at a soil to water ratio of 1:2.5. Soil organic matter (SOM) was determined using the potassium dichromate external heating method. Total nitrogen (TN) was quantified by the Kjeldahl digestion method. Total phosphorus (TP) was measured via sodium hydroxide fusion combined with the molybdenum antimony ascorbic acid colorimetric method. Total potassium (TK) was determined by sodium hydroxide fusion flame photometry. Dissolved organic carbon (DOC) was analyzed by water extraction, membrane filtration, and a total organic carbon (TOC) analyzer. Hydrolyzable nitrogen (HN) was assayed by the alkaline diffusion method. Available phosphorus (AP, for acidic soils) was extracted with hydrochloric acid ammonium fluoride and determined using the molybdenum antimony ascorbic acid colorimetric method. Available potassium (AK) was extracted with ammonium acetate and measured by flame photometry. Cation exchange capacity (CEC) was determined by the ammonium acetate exchange method (Xu et al., 2024).

Soil sucrase (SC) activity was assayed using the 3,5 dinitrosalicylic acid colorimetric method. Urease (UE) activity was determined by the sodium phenate sodium hypochlorite colorimetric method. Acid phosphatase (ACP) activity was measured colorimetrically using disodium phenyl phosphate as the substrate. Catalase (CAT) activity was quantified by the hydrogen peroxide UV absorption method. The activities of β-glucosidase (β-GC) and N-acetyl-β-D-glucosaminidase (NAG) were both determined using the p nitrophenyl substrate assay (Cui et al., 2022).

### 2.5 Amplicon sequencing and taxonomic annotation of soil samples

Prior to sequencing, soil DNA was extracted using the E.Z.N.A. DNA Kit (Omega Bio Tek, Norcross, GA, USA). The DNA concentration and purity were assessed with a NanoDrop 2000 spectrophotometer (Thermo Fisher Scientific Inc., MA, USA). Qualified DNA samples were subjected to subsequent PCR amplification. Target fragments were amplified using barcode tagged specific primers. The V3 V4 region of the bacterial 16S rRNA gene was amplified with primers 338F (5′ ACTCCTACGGGAGGCAGCAG 3′) and 806R (5′ GGACTACHVGGGTWTCTAAT 3′). For fungi, the ITS1 region was amplified using primers ITS1F (5′ CTTGGTCATTTAGAGGAAGTAA 3′) and ITS2R (5′ GCTGCGTTCTTCATCGATGC 3′). PCR products were verified by 2 % agarose gel electrophoresis, gel purified with the AxyPrep DNA Kit (Axygen Biosciences, Union City, CA, USA), and eluted with Tris HCl buffer. Purified amplicons were accurately quantified using the QuantiFluor™ ST Blue Fluorescence Quantification System (Promega, Madison, WI, USA). Amplicons were pooled in equal molar ratios for library construction. High throughput sequencing was performed on the Illumina HiSeq 2500 platform by Majorbio Bio Pharm Technology Co., Ltd., Shanghai, China. Sequence data generated in this study have been deposited in the National Center for Biotechnology Information (NCBI), with bacterial sequences under BioProject accession PRJNA1520269 and fungal sequences under PRJNA1520276.

The optimized sequences were clustered into operational taxonomic units (OTUs) at a 97 % sequence similarity cutoff using Uparse software (version 7.0.1090). Taxonomic classification of representative OTU sequences was performed via the Bayesian algorithm implemented in RDP classifier (version 2.11). The SILVA Release 138 database and UNITE Release 8.0 database were used for taxonomic annotation of bacteria and fungi, respectively, with a confidence threshold set at 0.70.

### 2.6 Data analysis

Raw data were organized using Microsoft Excel 2021. Independent field plots were treated as the basic experimental units for statistical analysis, with n = 4 for each cropping system. Soil chemical properties, enzyme activities, and α-diversity indices were compared between CK and DM using Welch’s t-test. Differential abundance at the genus level was analyzed using Welch’s t-test, with false discovery rate (FDR) correction performed using the Benjamini–Hochberg procedure; P < 0.05 was considered statistically significant. Amplicon data were normalized to a uniform sequencing depth. Principal coordinate analysis (PCoA) was performed based on Bray–Curtis distances, and differences in community composition were tested using PERMANOVA (adonis2, 999 permutations). PERMDISP (betadisper and permutest, 999 permutations) was also used to assess multivariate within-group dispersion and aid interpretation of PERMANOVA results. PERMANOVA results are reported as degrees of freedom, pseudo-F statistic, R^2^, and permutation-based P value. Mantel tests were used to evaluate overall associations between microbial communities and soil environmental factors, using Bray–Curtis distances for microbial communities and Euclidean distances for environmental variables; statistical significance was evaluated using Spearman correlation with 999 permutations. For the major enriched microbial taxa identified by screening, Spearman rank correlation analysis was further used to evaluate associations between their relative abundances and soil chemical properties and enzyme activities. Statistical analyses and graphical visualization were performed using R, GraphPad Prism v10.1.2, and Origin 2024.

### 2.7 Isolation and functional verification of soil culturable bacteria

#### 2.7.1 Culture media

Isolation media included Nutrient Agar (NA) medium, Tryptic Soy Agar (TSA) medium, and Reasoner’s 2A (R2A) medium. Functional screening media contained lignin medium, cellulose medium, casein medium, Ashby nitrogen free medium, organic phosphate solubilizing medium, inorganic phosphate solubilizing medium, feldspar based potassium solubilizing medium, zinc solubilizing medium, and iron free Chrome Azurol S (CAS) medium (Shi and Liu, 2026; Wang N. et al., 2024).

#### 2.7.2 Bacterial isolation, 16S rRNA-based identification and bioactivity screening

Bacterial isolation and purification: Two grams of soil sample were added into an Erlenmeyer flask containing 198 mL of sterile water, and shaken at 180 r/min and 30 °C for 4–6 h to obtain a well-homogenized soil suspension. Serial dilutions of 10^−4^, 10^−5^, and 10^−6^ were prepared from the soil suspension. An aliquot of 200 μL of each dilution was spread onto isolation-medium plates with three replicates per dilution. Plates were incubated at 30 °C for 48 h. Single colonies with distinct morphological characteristics were picked, inoculated onto corresponding agar slants, and preserved in glycerol at −80 °C.

Strain identification: Fifty microliters of 10% (w/v) Chelex-100 solution was transferred to a 1.5 mL centrifuge tube. Well-developed single colonies were picked from agar plates with an inoculating loop and fully suspended by agitation in the Chelex-100-containing solution. The suspension was subjected to a 20 min water-bath incubation at 100 °C. Upon cooling to room temperature, the suspension was centrifuged at 12 000 rpm for 5 min, and the resultant supernatant was harvested and adopted as the PCR template. The 16S rDNA fragment was amplified by PCR employing universal forward primer 27F (5′-AGAGTTTGATCCTGGCTCAG-3′) and reverse primer 1492R (5′-GGTTACCTTGTTACGACTT-3′). Detailed PCR reaction components and amplification conditions are provided in **Supplementary Table S1**. Amplified products were separated by 1% agarose gel electrophoresis, followed by gel purification and recovery. Purified PCR amplicons were sent to Tsingke Biotechnology Co., Ltd. (Beijing, China) for Sanger sequencing. Homology searches were conducted against the EzBioCloud database (www.ezbiocloud.net). A phylogenetic tree was built in MEGA 7.0 software, and 1 000 bootstrap replicates were performed to assess the taxonomic affiliation of the isolates.

Bioactivity assays (Shi and Liu, 2026; Wang N. et al., 2024): The strains to be tested were spot-inoculated onto the corresponding functional agar plates and incubated in the dark at 28 °C for 48 h. Phenotypes examined included growth on nitrogen-free medium, inorganic phosphorus solubilization, organic phosphorus transformation, potassium solubilization, zinc solubilization, siderophore production, and utilization of lignin, cellulose, and protein substrates. Plates used to assess cellulolytic activity were stained with 0.1% Congo red for 30 min and destained with 1 mol L^−1^ NaCl for 30 min before hydrolysis zones were observed. The functional index was calculated as SI = D/d, where D is the total diameter from the outer edge on one side of the hydrolysis/clear zone to the outer edge on the opposite side, and d is the colony diameter. Three parallel replicates were established for each treatment.

#### 2.7.3 Determination of bacterial growth curve and strain compatibility

The bacterial growth curve was determined by the turbidimetric method. A revived single colony was inoculated into liquid R2A medium and incubated with shaking at 28 °C until the strain reached the logarithmic growth phase to prepare the seed culture. The seed culture was inoculated into fresh liquid R2A medium at an inoculation ratio of 1 %. Samples were collected immediately after inoculation, and the initial optical density at 600 nm (OD_600_) was measured using a microplate reader. The culture system was incubated on a constant-temperature shaker with continuous agitation. Samples were collected at regular time intervals for OD_600_ measurement until OD_600_ values became stable or began to decline. The bacterial growth curve was plotted with incubation time on the x-axis and corresponding OD_600_ values on the y-axis.

Strain compatibility was assayed using the double-layer agar Oxford-cup method. A 2 % water-agar base layer was prepared in 9 cm-diameter sterile Petri dishes. Six sterilized Oxford cups were placed vertically on the agar surface with equal spacing of 2.5 cm. The indicator strain was inoculated at 1 % (v/v) into R2A medium maintained at 45 °C. After thorough mixing, the bacterial-containing upper-layer medium was immediately poured onto the base agar. After solidification, the Oxford cups were removed to generate 6 mm-diameter sample wells. The challenging strains were adjusted to a cell suspension of 1 × 10^8^ CFU/mL, and 20 μL of each suspension was added into individual wells, with an equal volume of sterile water set as the control. Plates were incubated at 28 °C for 48 h. A clear inhibitory halo surrounding the well of the challenging strain indicated antagonism; otherwise, the strains were regarded as compatible. Each treatment was performed in triplicate (Du et al., 2022).

#### 2.7.4 Pot-Experiment for functional validation and measurement of activity related indices

Strain fermentation and inoculum preparation: Strains were inoculated into 500 mL Erlenmeyer flasks containing 200 mL of liquid R2A medium, and incubated with shaking at 180 r/min and 30 °C for 24–48 h. After fermentation, the bacterial culture was diluted with sterile distilled water to adjust the inoculum concentration to 1.0 × 10^8^ CFU/mL.

Test materials: The experimental soil was collected from continuously cropped tobacco-planting fields. The basic physicochemical properties of the soil before pot cultivation were as follows: pH 5.19, soil organic matter 62.74 g/kg, total nitrogen 2.56 g/kg, total phosphorus 1.33 g/kg, total potassium 20.64 g/kg, dissolved organic carbon 425.12 mg/kg, hydrolyzable nitrogen 274.68 mg/kg, available phosphorus 306.02 mg/kg, and available potassium 452.05 mg/kg. The tobacco cultivar used in this study was Yunyan 87.

Experimental design: Five treatments were set up: CK1 (sterile water application), CK2 (sterile liquid R2A medium application), T1 (*Sphingomonas* inoculum), T2 (*Burkholderia-Caballeronia-Paraburkholderia* inoculum), and T3 (1:1 mixed inoculum of the above-mentioned two genera). Each treatment contained ten biological replicates.

Measurements and methods: Plants and rhizosphere soil were collected 50 d after flue-cured tobacco planting. Plant height, stem circumference, and fresh/dry weights of shoots and roots were measured. Total nitrogen, total phosphorus, and total potassium concentrations in shoots and roots were determined according to NY/T 2017—2011. Soil SOM, TN, TP, TK, DOC, HN, AP, and AK were also measured using the methods described in Section 2.4. The pot experiment included 10 biological replicates per treatment (n = 10). Differences among treatments were evaluated using one-way analysis of variance (one-way ANOVA), followed by Tukey’s honestly significant difference (HSD) test for *post hoc* pairwise comparisons. Statistical significance was set at *P* < 0.05.

## 3 Results and analysis

### 3.1 Effects of tobacco-barley rotation on soil chemical properties

Compared with CK, the DM treatment significantly increased soil pH, SOM, TK, DOC, HN, AP and AK by 0.96 %, 3.80 %, 3.88 %, 19.65 %, 5.90 %, 3.23 %, and 5.45 %, respectively (*P* < 0.05). No significant differences (*P* > 0.05) among treatments were observed for soil TN, TP and CEC (**Figure 1**). These results indicated that the DM treatment effectively improved the chemical properties of tobacco-growing soil and facilitated the activation and accumulation of soil available nutrients.

**Figure 1:**
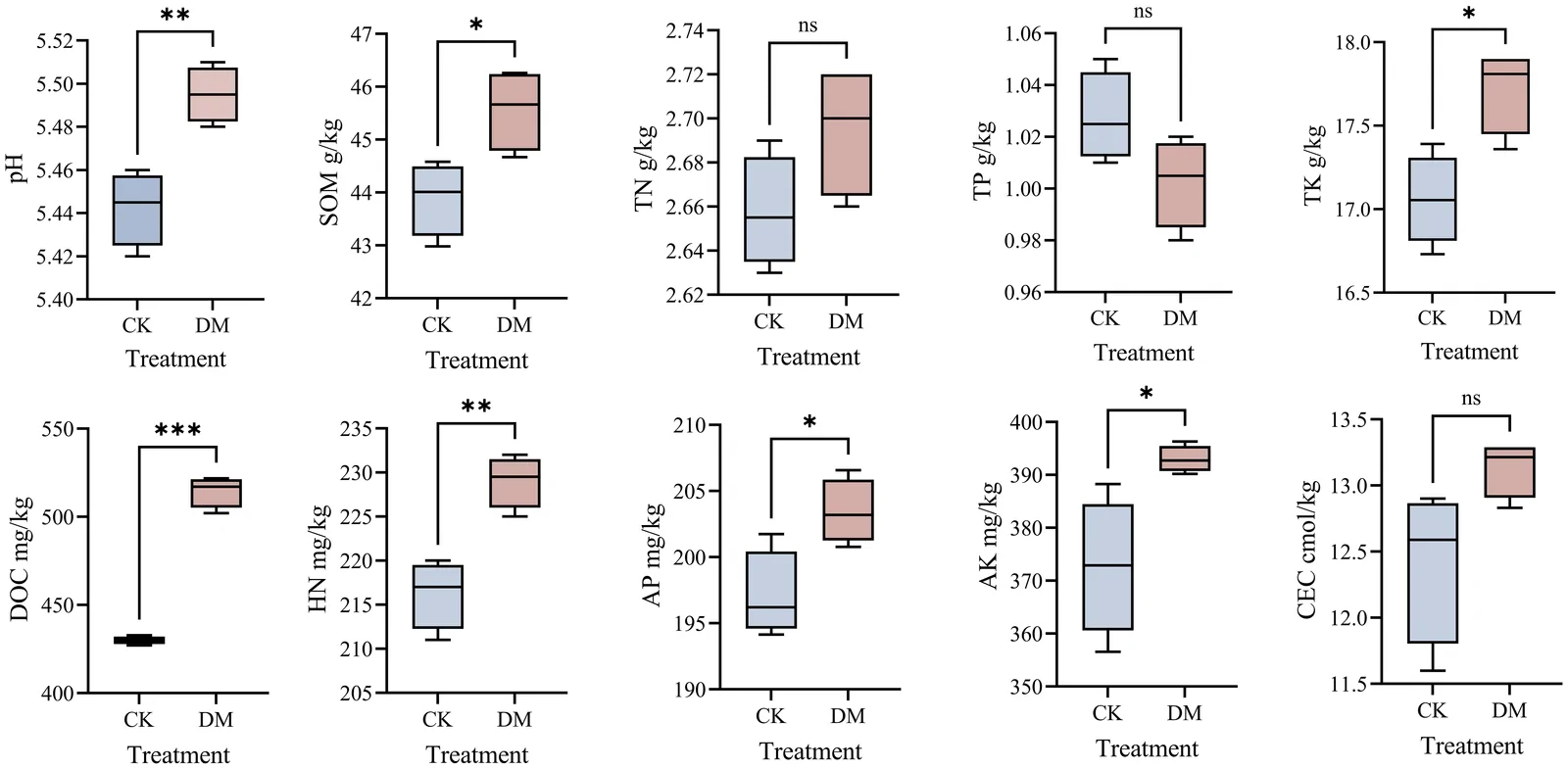
Effects of barley rotation on soil chemical properties. CK, flue-cured tobacco–fallow; DM, flue-cured tobacco–barley rotation. Each treatment contained four independent field plots (n = 4). The center line of each boxplot represents the median, and the boxes and whiskers indicate the data distribution. SOM, soil organic matter; TN, total nitrogen; TP, total phosphorus; TK, total potassium; DOC, dissolved organic carbon; HN, hydrolyzable nitrogen; AP, available phosphorus; AK, available potassium; CEC, cation exchange capacity. CK and DM were compared using Welch’s t-test. *** indicates *P* < 0.001, ** indicates 0.001 ≤ *P* < 0.01, * indicates 0.01 ≤ *P* < 0.05, and ns indicates no significant difference.

### 3.2 Effects of tobacco barley rotation on soil enzyme activities

Compared with CK, the DM treatment significantly enhanced the activities of multiple key hydrolytic enzymes in tobacco growing soil (**Figure 2**). The activities of carbon cycling related SC and β GC were significantly increased by 23.80 % and 66.28 %, respectively (*P* < 0.05). The activity of ACP involved in phosphorus cycling was significantly increased by 15.23 %. NAG, which participated in chitin degradation, exhibited the highest increment, reaching 176.32 %. No significant differences (*P* > 0.05) in UE related to nitrogen cycling (urea hydrolysis) and CAT associated with oxidative stress regulation were observed between the two rotation patterns.

**Figure 2:**
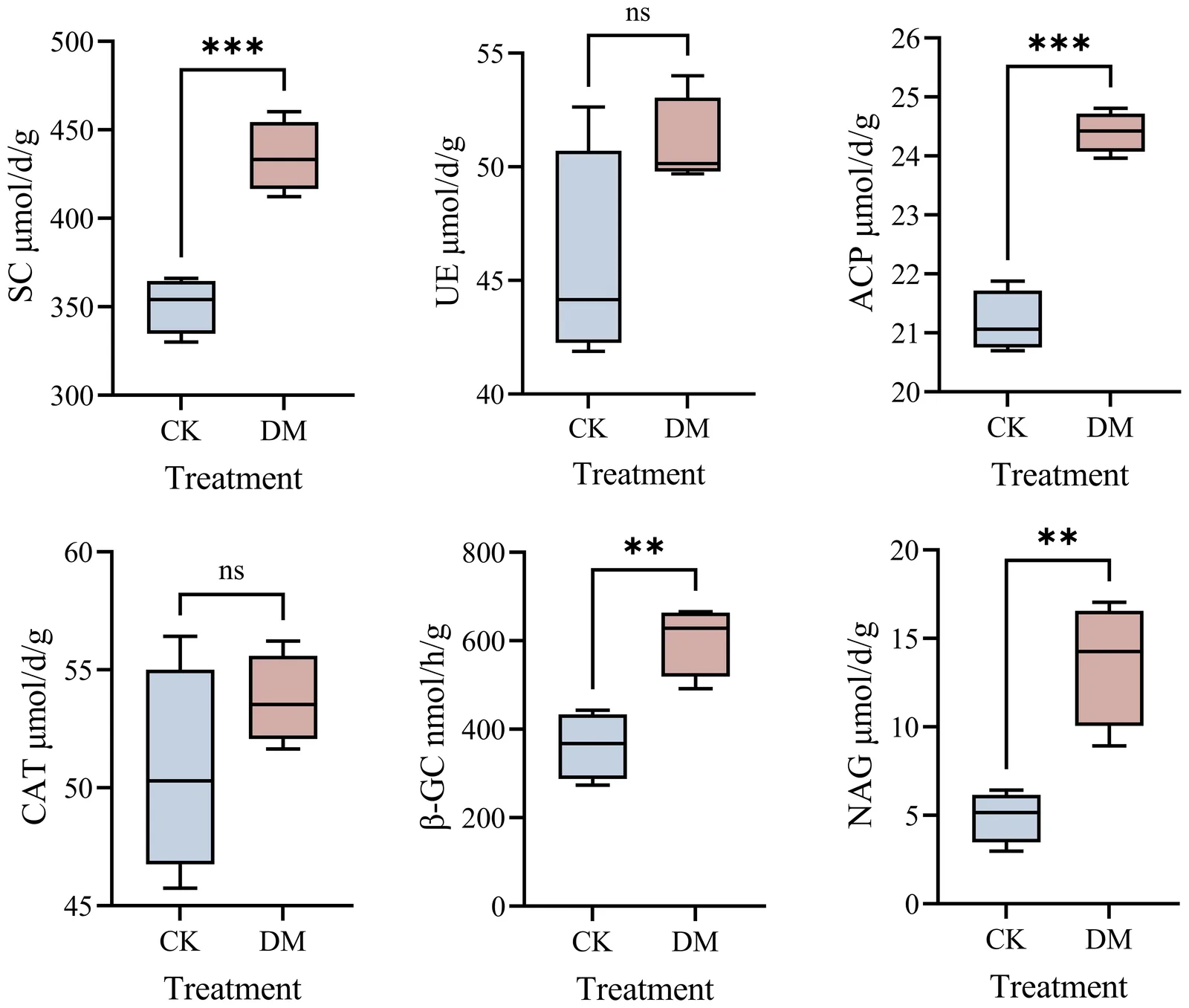
Effects of barley rotation on soil enzyme activities. CK, flue-cured tobacco–fallow; DM, flue-cured tobacco–barley rotation. Each treatment contained four independent field plots (n = 4). The center line of each boxplot represents the median, and the boxes and whiskers indicate the data distribution. SC, sucrase; UE, urease; ACP, acid phosphatase; CAT, catalase; β-GC, β-glucosidase; NAG, N-acetyl-β-D-glucosaminidase. CK and DM were compared using Welch’s t-test. *** indicates *P* < 0.001, ** indicates 0.001 ≤ *P* < 0.01, and ns indicates no significant difference.

### 3.3 Responses of soil microbial diversity to tobacco-barley rotation

Bacterial and fungal α diversity showed contrasting responses under the two cropping systems (**Figures 3A, C**). Compared with CK, DM decreased the bacterial Shannon, Chao1, and ACE indices by 8.35%, 17.50%, and 18.79%, respectively (*P* < 0.05), while increasing the Simpson index by 66.30%. In contrast, the fungal Shannon, Chao1, and ACE indices increased by 28.00%, 9.58%, and 9.84%, respectively, whereas the Simpson index decreased by 67.14%. Thus, barley rotation did not uniformly increase α diversity across all microbial groups; instead, it produced a group-specific response characterized by reduced bacterial richness/evenness and increased fungal diversity.

**Figure 3:**
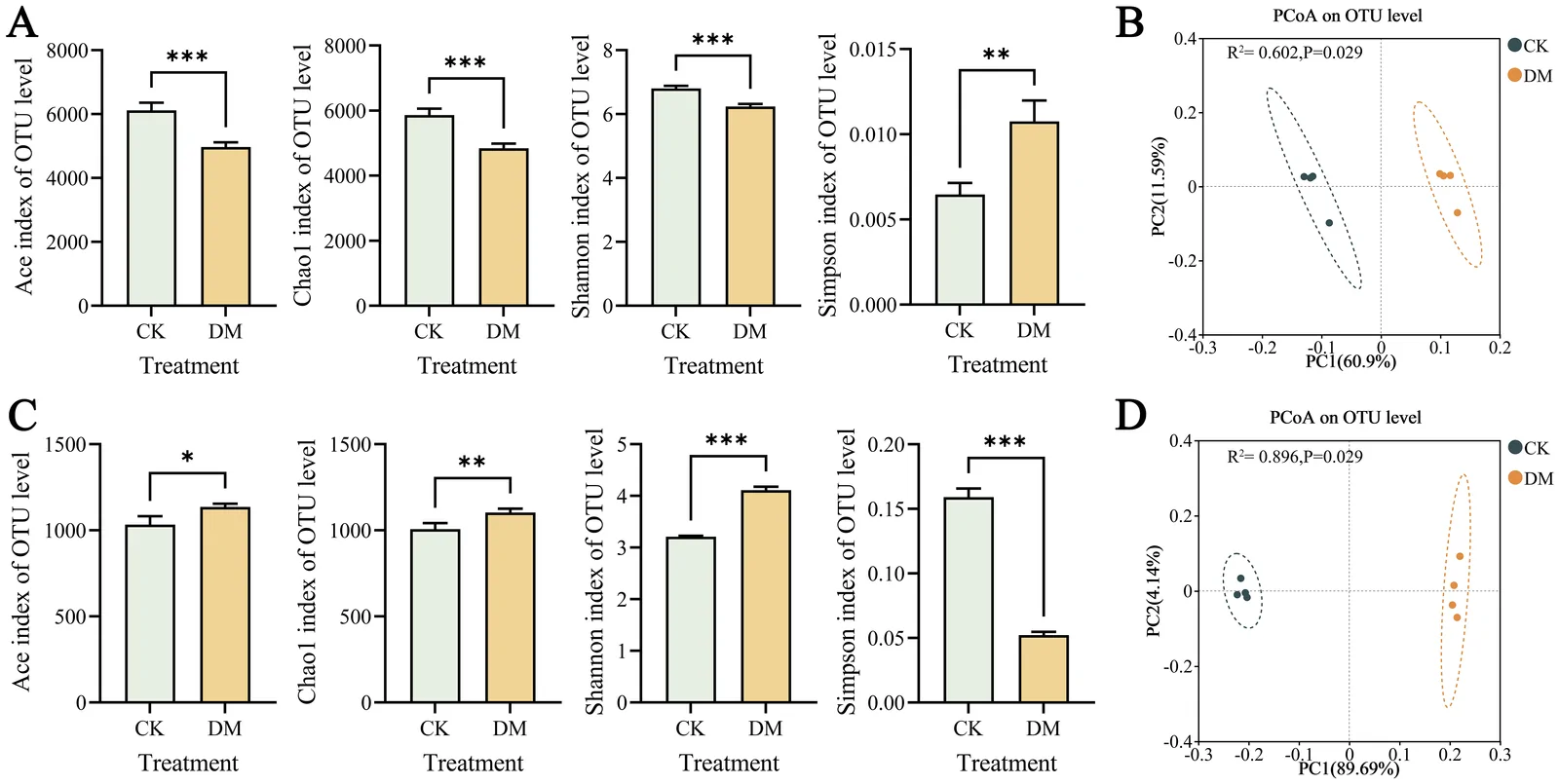
Effects of tobacco-barley rotation on soil microbial diversity. CK, flue-cured tobacco–fallow; DM, flue-cured tobacco–barley rotation; each treatment comprised n = 4 independent field plots. Bars represent means and error bars represent SD. **(A, C)** show bacterial and fungal α diversity, respectively; **(B, D)** show bacterial and fungal PCoA based on Bray–Curtis distances, respectively. α diversity was analyzed using Welch’s t-test; β diversity was analyzed using PERMANOVA (999 permutations), with PERMDISP used to assess within-group dispersion. *** indicates *P* < 0.001, ** indicates 0.001 ≤ *P* < 0.01, and * indicates 0.01 ≤ *P* < 0.05.

PCoA based on Bray–Curtis distances further showed clear separation of both bacterial and fungal community compositions between CK and DM (**Figures 3B, D**). The first two PCoA axes explained 72.49% of the total variation in bacterial communities and 93.83% in fungal communities. PERMANOVA further indicated a significant difference in bacterial community structure between CK and DM (pseudo-F = 9.063, R^2^ = 0.602, *P* = 0.029). The corresponding PERMDISP analysis detected no significant difference in within-group dispersion between the two treatments (F = 1.267, P = 0.257), indicating that the bacterial PERMANOVA result mainly reflected differences in community composition between treatments. PERMANOVA likewise showed a significant difference in fungal communities between CK and DM (pseudo-F = 51.715, R^2^ = 0.896, P = 0.029). However, fungal PERMDISP was also significant (F = 34.296, P = 0.029), indicating that within-group dispersion differed between treatments. Therefore, the fungal PERMANOVA result should be interpreted cautiously, as its significance may have been influenced by both differences in community centroid location and within-group heterogeneity. Complete statistical results are provided in **Supplementary Table**.

### 3.4 Responses of soil microbial community structure to tobacco-barley rotation

Analysis of soil microbial community structure indicated that barley rotation substantially altered both bacterial and fungal community compositions (**Figure 4**). At the phylum level for bacteria, Pseudomonadota and Acidobacteriota were the dominant phyla in both CK and DM treatments. Their relative abundances increased from 36.75 % to 42.21 % and from 19.88 % to 22.04 %, respectively, under DM treatment compared with CK (**Figure 4A**). At the genus level, the DM treatment significantly increased the relative abundances of *Sphingomonas*, *Pseudolabrys*, and *Burkholderia-Caballeronia-Paraburkholderia* by 26.16 %-61.15 %, whereas it significantly decreased the relative abundances of *Flavobacterium*, *MND1*, and *Neobacillus* by 29.23 %-50.27 % (*P *< 0.05, **Figures 4B, C**). For the fungal community at the phylum level, compared with CK, the relative abundances of Ascomycota, Basidiomycota, and Mortierellomycota in DM treatment increased from 47.43 % to 62.75 %, 6.16 % to 15.33 %, and 6.95 % to 13.12 %, respectively. By contrast, the relative abundance of *Chytridiomycota* decreased from 37.42 % to 2.67 % (**Figure 4D**). At the genus level, the relative abundances of *Mortierella*, *Cladosporium*, and *Saitozyma* significantly increased by 128.18 %-707.07 %, while those of *Albifimbria* and *Lobulomycetales_gen_Incertae_sedis* significantly decreased by 73.13 % and 98.84 %, respectively (**Figures 4E, F**).

**Figure 4:**
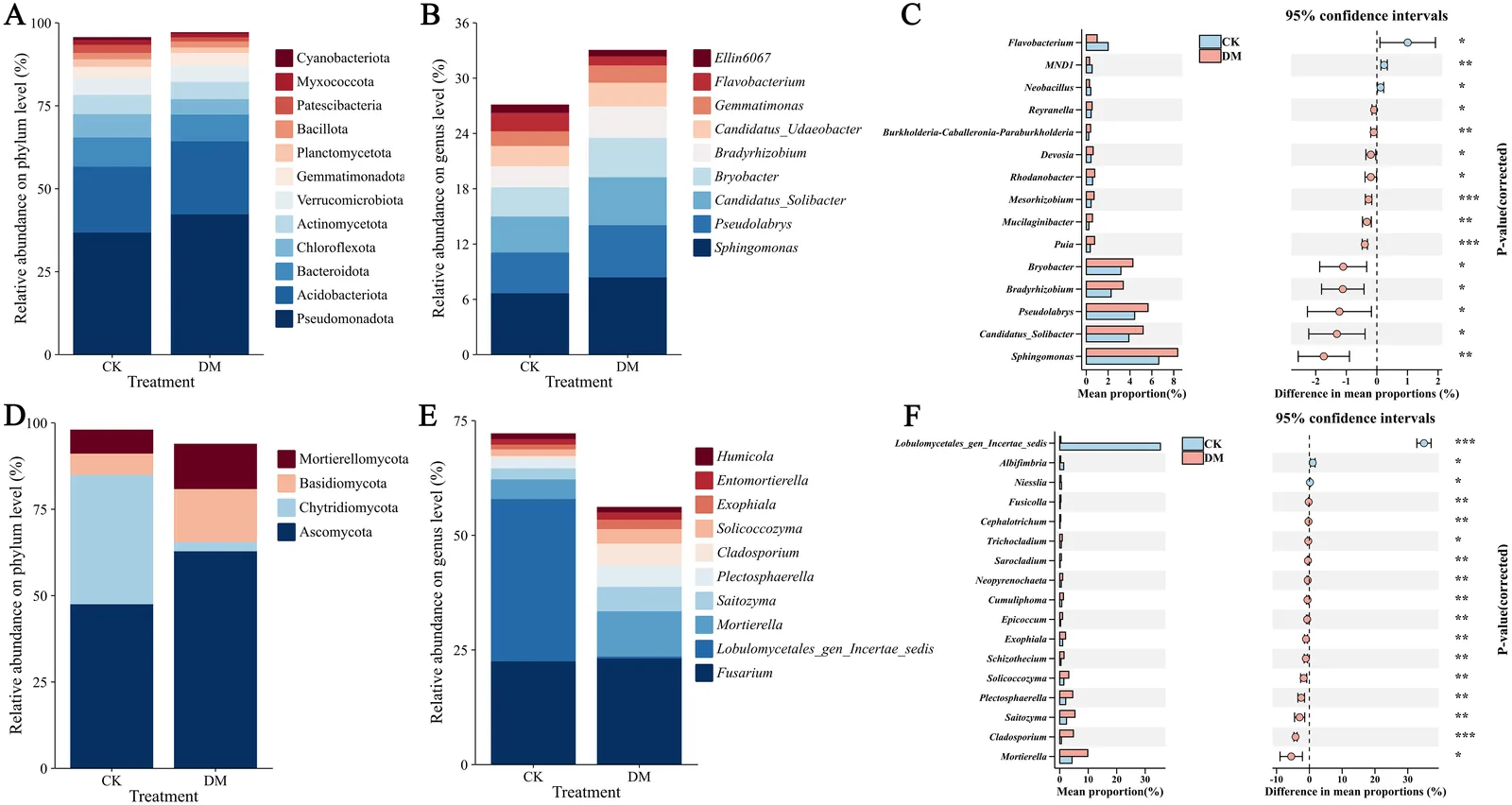
Effects of tobacco-barley rotation on soil microbial community composition. CK, flue-cured tobacco–fallow; DM, flue-cured tobacco–barley rotation; each treatment comprised n = 4 independent field plots. **(A)** relative abundance of bacterial phyla (>1%); **(B)** relative abundance of bacterial genera (>1%); **(C)** differential abundance analysis of dominant bacterial genera (top 30); **(D)** relative abundance of fungal phyla (>1%); **(E)** relative abundance of fungal genera (top 10); **(F)** differential abundance analysis of dominant fungal genera (top 30). Genus-level differences were analyzed using Welch’s t-test with Benjamini–Hochberg FDR correction (*P* < 0.05). *** indicates *P* < 0.001, ** indicates 0.001 ≤ *P* < 0.01, and * indicates 0.01 ≤ *P* < 0.05.

### 3.5 Candidate enriched microbial taxa and their associations with soil environmental factors

Mantel tests showed significant statistical associations between soil microbial community composition and multiple soil chemical properties and enzyme activity variables (**Figure 5A**). Spearman correlation analysis was further performed between microbial taxa showing significant differential abundance and soil environmental factors (**Figure 5B**). Under DM, *Sphingomonas* had a relatively high abundance and was significantly correlated with multiple soil variables, including TN, AP, HN, β-GC, DOC, AK, SOM, and TK. *Burkholderia-Caballeronia-Paraburkholderia* was likewise enriched under DM and significantly correlated with ACP, HN, SC, β-GC, DOC, AK, SOM, and TK. Among fungi, *Schizothecium*, *Cladosporium*, *Trichocladium*, and *Neopyrenochaeta* were enriched under DM and showed significant associations of varying magnitude with environmental factors including NAG, SOM, ACP, HN, and AP (**Figure 5C**). Integrating the differential-abundance and environmental-association results, *Sphingomonas* and *Burkholderia-Caballeronia-Paraburkholderia* simultaneously met the following criteria: relative enrichment under DM, membership among relatively abundant differential bacterial taxa, and significant statistical associations with multiple soil nutrient or enzyme activity variables. Therefore, these two taxa were defined as “candidate enriched bacterial taxa” for subsequent cultivation, isolation, and functional screening.

**Figure 5:**
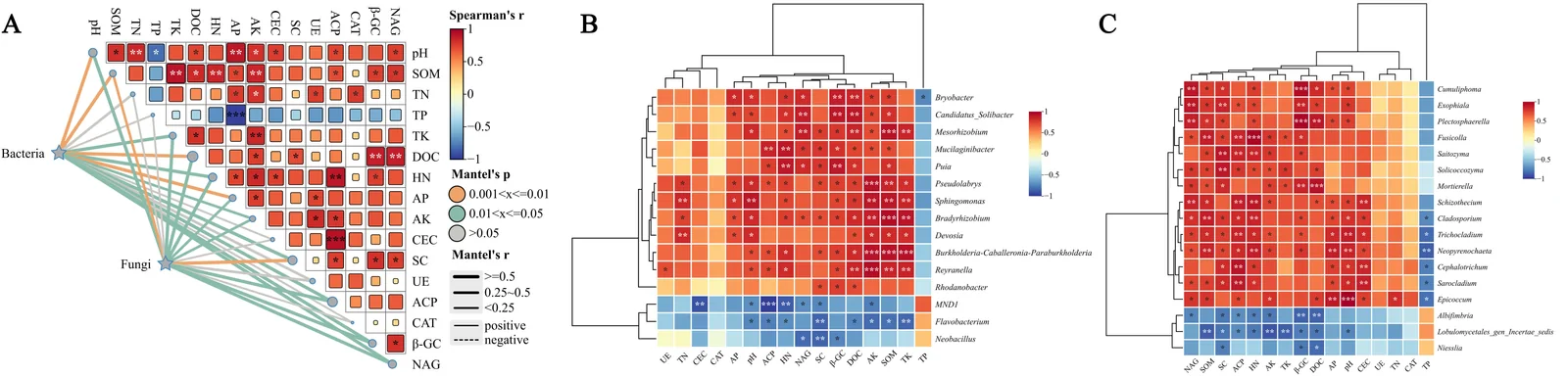
Relationships between environmental factors and soil microbial communities. **(A)** Mantel test between microbial communities and environmental factors; **(B, C)** Spearman correlation analyses between differential bacterial and fungal taxa and environmental factors, respectively. *** indicates *P* < 0.001, ** indicates 0.001 ≤ *P* < 0.01, and * indicates 0.01 ≤ *P* < 0.05.

### 3.6 Isolation and preliminary taxonomic identification of culturable strains from candidate enriched taxa

A total of 255 culturable bacterial isolates were obtained using NA, TSA, and R2A media. Based on 16S rRNA gene sequence comparisons, these isolates were preliminarily assigned to 4 phyla and 64 genera (**Supplementary Figure S2**). By integrating the two candidate enriched bacterial taxa identified through amplicon sequencing, six related culturable isolates were obtained: DM9, DM30, DM31, DM53, DM67, and DM255 (**Figure 6**). The closest reference strains to DM9, DM30, and DM255 were *Burkholderia paludis* MSh1, *Burkholderia ambifaria* AMMD, and *Paraburkholderia graminis* C4D1M, with sequence similarities of 99.93%, 99.86%, and 99.43%, respectively. DM31, DM53, and DM67 were most closely related to *Sphingomonas sanxanigenens* DSM 19645, *Sphingomonas bisphenolicum* AO1, and *Sphingomonas oligophenolica* JCM 12082, with similarities of 98.98%, 99.49%, and 99.14%, respectively (**Supplementary Table S2**). In the phylogenetic tree, the six isolates clustered with different reference taxa, suggesting that at the level of preliminary 16S rRNA-based classification they were not repeated isolates of the same reference lineage (**Figure 7**). It should be noted that an operational taxonomic unit (OTU) is a clustering unit used in amplicon community analysis and is not appropriate for directly describing independently cultured isolates. Therefore, these six strains are not referred to as six OTUs in this study; high-resolution taxonomic relationships among the strains still require confirmation using whole-genome data.

**Figure 6:**
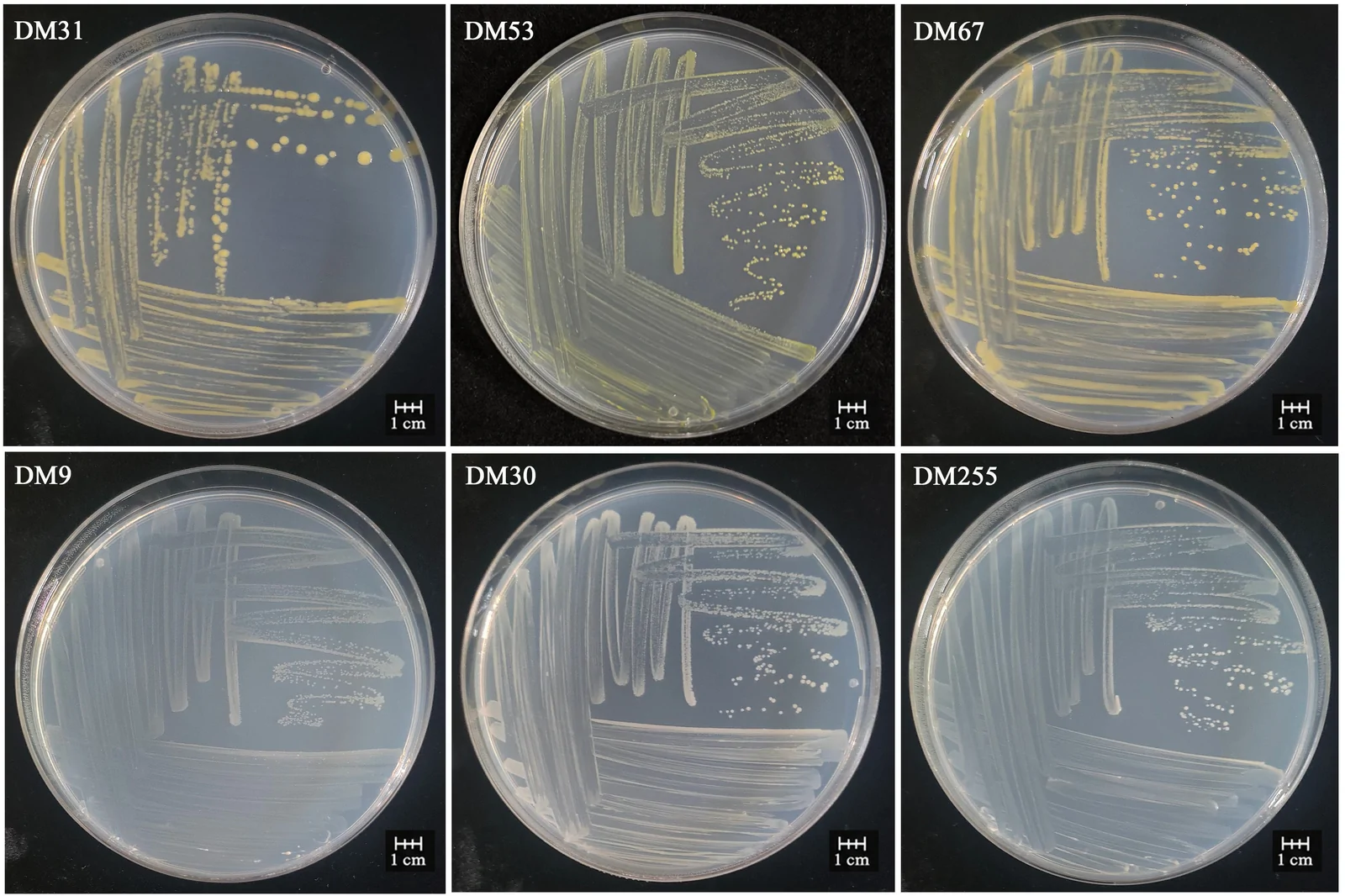
Colony-morphological characteristics of the six target isolates.

**Figure 7:**
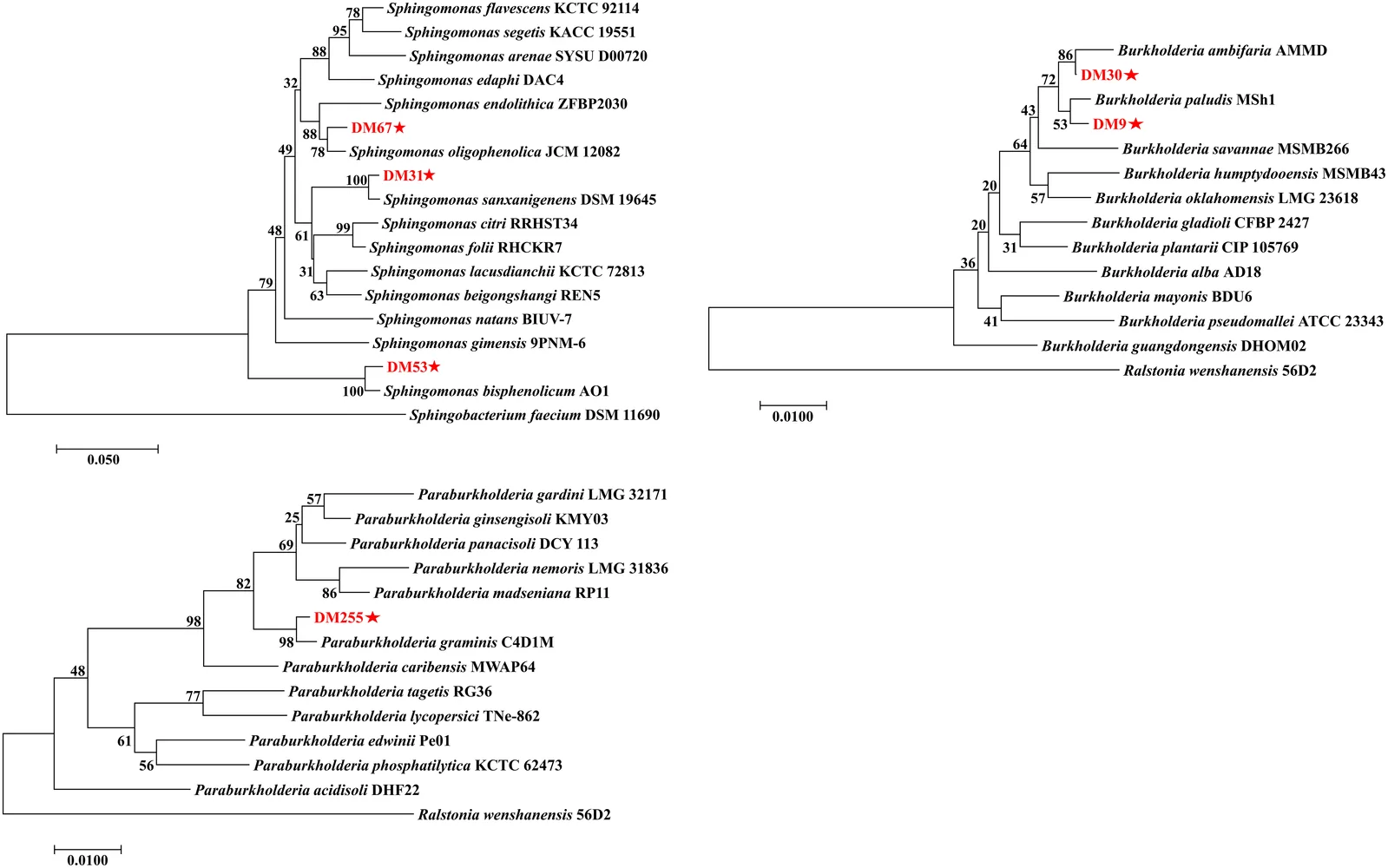
Neighbor-joining phylogenetic tree of target strains based on the 16S rDNA gene.

### 3.7 Functional phenotypes of candidate strains under culture conditions

To further evaluate the potential plant growth-promoting functions of the six isolates described above, multiple functional phenotypes were screened under culture conditions (**Figure 8**). Nine categories of phenotypes related to plant nutrient acquisition or substrate utilization were examined, including growth on nitrogen-free medium, inorganic phosphorus solubilization, organic phosphorus transformation, potassium solubilization, zinc solubilization, lignin degradation, cellulolytic enzyme activity, protein degradation, and siderophore production. Different strains exhibited different combinations of functions, indicating functional diversity among these isolates under culture conditions; detailed functional indices (SI) are provided in **Supplementary Table S3**. Growth-kinetic analysis showed that strains DM30, DM9, and DM255 reached maximum biomass at 16 h (OD_600_ = 0.445, 0.453, and 0.359, respectively), after which OD_600_ began to decline as the cultures entered the death phase. Strain DM53 reached its maximum biomass at 36 h (OD_600_ = 0.487), whereas DM31 and DM67 reached their maxima at 40 h (OD_600_ = 0.447 and 0.466, respectively), followed by similar declining trends (**Supplementary Figure S3A**). No distinct inhibition zones were observed using the double-layer agar Oxford cup method (**Supplementary Figure S3B**), indicating no antagonism among the six strains and good biocompatibility.

**Figure 8:**
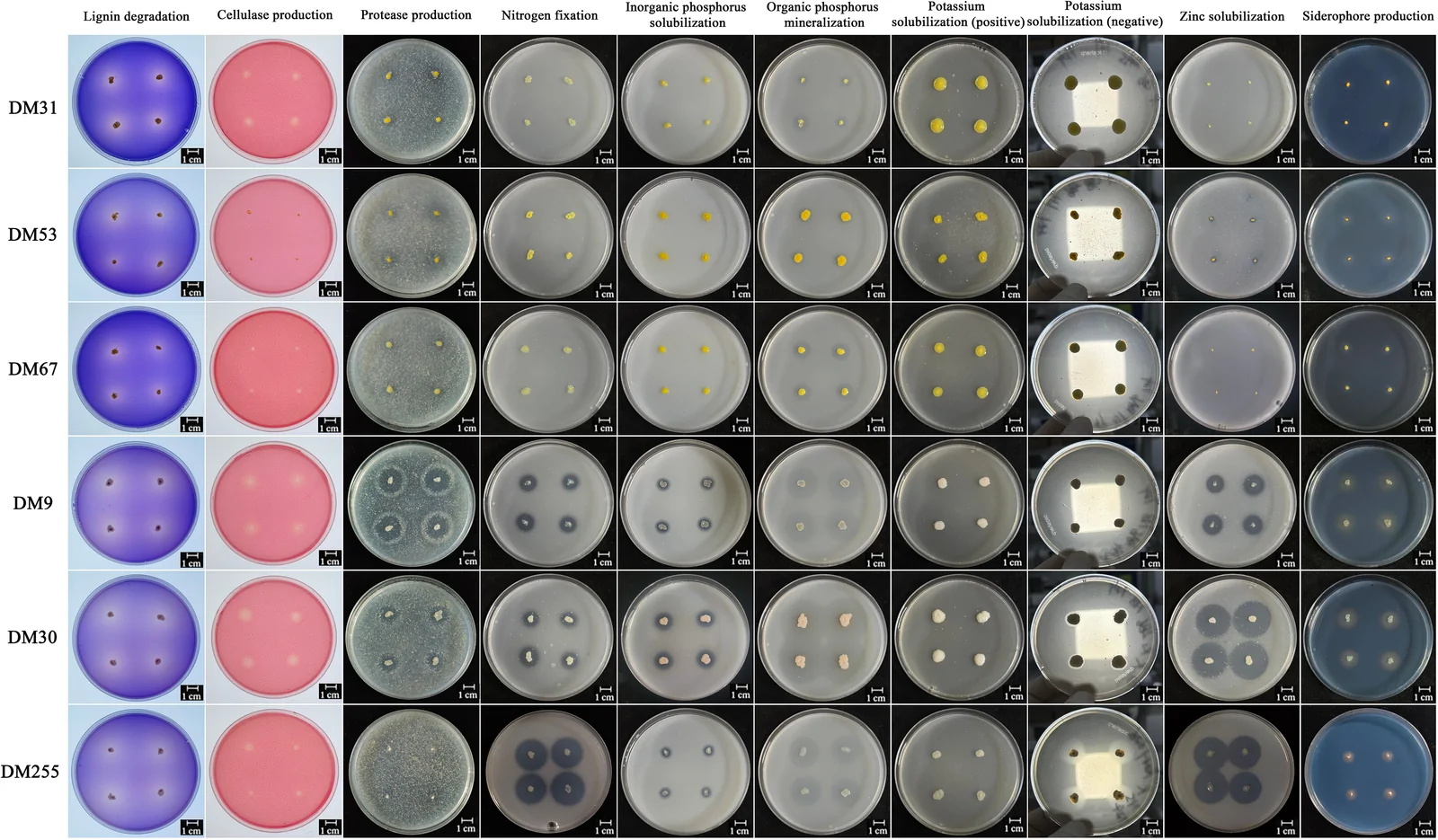
Qualitative *in vitro* functional phenotypes of the six target isolates.

### 3.8 Effects of candidate strains on flue-cured tobacco growth traits

Agronomic traits of flue-cured tobacco plants were measured on the 50th day after transplantation (**Figure 9A**). One-way analysis of variance (ANOVA) revealed that all agronomic traits under strain fermentation liquid treatments were significantly superior to those of the two control groups (*P *< 0.05). Compared with the CK1 treatment, the T1 treatment significantly increased plant height, stem circumference, above-ground fresh weight, root fresh weight, above-ground dry weight, and root dry weight by 24.39 %, 25.78 %, 88.40 %, 152.13 %, 115.53 %, and 116.17 %, respectively. For the T2 treatment, the corresponding indicators increased by 20.87 %, 25.33 %, 80.46 %, 118.41 %, 134.63 %, and 156.44 %. The T3 mixed-strain treatment produced the most prominent improvements in key growth and biomass indicators: stem circumference increased by 31.69 %, above-ground dry weight and root dry weight increased by 168.49 % and 179.21 %, respectively. Plant height, above-ground fresh weight, and root fresh weight were also significantly increased by 23.94 %, 86.30 %, and 141.96 %, relative to CK1 (**Figure 9B**). These results indicate that the candidate strains have the potential to promote flue-cured tobacco growth under controlled greenhouse conditions; however, equivalent effects in the field cannot be inferred from these results.

**Figure 9:**
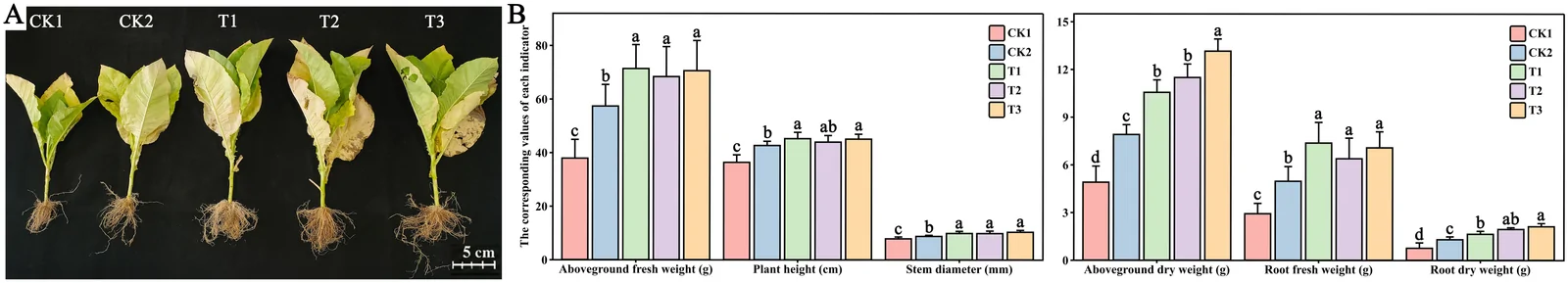
Phenotypic characteristics **(A)** and agronomic trait analysis **(B)** of flue-cured tobacco plants after 50 days of treatment with different strains. Each treatment included 10 biological replicates (n = 10). Different letters indicate significant differences (*P* < 0.05, Tukey HSD test). Bars represent means and error bars represent SD.

### 3.9 Effects of core strains on element contents of flue-cured tobacco plants and chemical properties of tobacco-planted soil

The total nitrogen, total phosphorus, and total potassium contents of flue-cured tobacco plants were determined on the 50th day after transplantation. The results showed that no significant differences in element contents were observed between the CK1 and CK2 control groups (*P* > 0.05), indicating that the R2A medium itself exerted no significant influence on plant element accumulation. The inoculation treatments T1, T2, and T3 significantly increased nitrogen, phosphorus, and potassium contents in both above-ground parts and roots of flue-cured tobacco plants (*P* < 0.05). Among them, the T3 mixed-strain treatment exhibited the most prominent effects. The TN, TP, and TK contents in above-ground tissues reached 11.85 ± 1.23 g/kg, 3.79 ± 0.26 g/kg, and 60.20 ± 3.97 g/kg, which were significantly increased by 29.48 %, 29.83 %, and 39.02 %, respectively, compared with CK1. The corresponding element contents in roots were 11.95 ± 0.29 g/kg, 3.79 ± 0.05 g/kg, and 29.78 ± 2.66 g/kg, with increases of 16.92 %, 19.25 %, and 29.93 % (**Figure 10**). Although T1 and T2 treatments also elevated plant element contents, their effects were generally lower than those of T3. These findings suggested synergistic effects among these strains.

**Figure 10:**
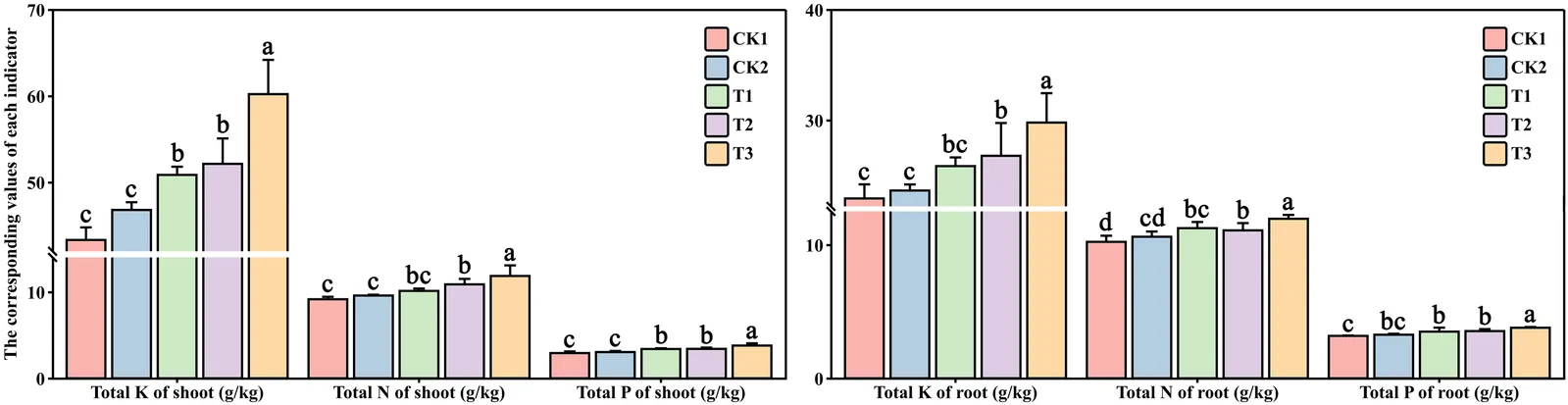
Analysis of element contents in flue-cured tobacco plants after 50 days of treatment with different strains. Each treatment included 10 biological replicates (n = 10). Different letters indicate significant differences (*P* < 0.05, Tukey HSD test). Bars represent means and error bars represent SD.

The rhizosphere soil chemical indicators of each treatment were determined on the 50th day after transplantation, and the results were presented in **Supplementary Figure S4**. No significant differences in soil SOM, TN, TP, TK, and HN contents were observed across all treatments (*P* > 0.05). Nevertheless, soil DOC and AK contents in the inoculation treatments (T1, T2, T3) were significantly lower (*P* < 0.05) than those in the two control groups (CK1 and CK2). The T3 mixed-strain treatment exhibited the highest AP content, which was significantly higher than that of all other treatments. These changes occurred concurrently with increased plant nutrient accumulation after inoculation. However, decreases or increases in soil available nutrients may be jointly influenced by plant uptake, microbial metabolism, and soil chemical equilibria; therefore, these observations alone cannot establish that the candidate strains directly drove soil nutrient cycling.

## 4 Discussion

Soil quality degradation caused by long-term continuous cropping is an important constraint on sustainable tobacco production. By changing crop type, root activity, and patterns of organic matter input, crop rotation can exert integrated effects on soil physicochemical properties and biological processes (Ceotto and Castelli, 2002). Based on a three-year continuous field experiment, this study found that, compared with the flue-cured tobacco–fallow system, flue-cured tobacco–barley rotation significantly increased soil pH and improved multiple indicators of soil nutrient availability. The increase in dissolved organic carbon (DOC) was the most pronounced; available phosphorus, available potassium, and hydrolyzable nitrogen also increased significantly, whereas total nitrogen and total phosphorus showed no significant changes. These findings indicate that barley rotation primarily altered the active carbon pool and nutrient availability in soil without markedly changing the total pools of some nutrients. Both barley root exudation and residue decomposition may increase inputs of bioavailable organic carbon, which could explain the increase in DOC. Meanwhile, organic acids generated during residue decomposition, ion exchange, and microbial metabolic activity may promote the transformation of some soil phosphorus and potassium from less available to more available forms (Gan and Drinkwater, 2025; Rezaeinejad et al., 2021). In addition, as a gramineous crop with a distinct growth period and root traits, barley may alter the temporal patterns of soil nutrient uptake and return. Therefore, the observed pattern of “increased available nutrients while some total nutrient pools remained stable” more likely reflects changes in nutrient turnover rates and availability under the rotation system rather than a synchronous increase in the total soil nutrient pool.

Changes in soil enzyme activities further support an association between barley rotation and altered soil biochemical processes. DM significantly increased the activities of sucrase, β-glucosidase, acid phosphatase, and N-acetyl-β-D-glucosaminidase (NAG), whereas urease and catalase showed no significant changes. Sucrase and β-glucosidase participate in the degradation of labile organic carbon and cellulose, and their increased activities were consistent with the higher DOC concentration, suggesting that barley roots and residue inputs may have enhanced microbial metabolic processes associated with active carbon transformation (Bandick and Dick, 1999; Borase et al., 2020; Peters and Thiele-Bruhn, 2022). The concurrent increase in acid phosphatase activity and available phosphorus also suggests that phosphorus mineralization may have been more active under rotation (Trasar-Cepeda et al., 2008; Cai et al., 2015). NAG mainly participates in the decomposition of chitin and other nitrogen-containing organic matter, and its marked increase indicates a change in the potential for transformation of nitrogen-containing organic matter. Nevertheless, synchronous changes in soil enzyme activities and nutrient concentrations remain process-level associative evidence and cannot establish that a specific microbial taxon directly caused the corresponding nutrient changes. The specific microbially driven processes require further validation using approaches such as functional gene analysis, transcript-level measurements, or stable-isotope tracing.

Soil microorganisms are central components of soil ecosystems, and their community structure and diversity respond sensitively to cropping practices, making them important indicators of soil health (Smith et al., 2015). This study found that microbial communities exhibited clear group-specific responses to barley rotation. Compared with CK, DM reduced the bacterial Shannon, Chao1, and ACE indices but increased the corresponding diversity indices of the fungal community. This result indicates that rotation does not necessarily increase α diversity simultaneously across different microbial groups; rather, its effects may be jointly shaped by the type of resource input, soil environmental conditions, and the life-history strategies of different microorganisms. For bacteria, reduced diversity may reflect stronger environmental filtering under barley rotation, allowing taxa capable of rapidly exploiting root exudates and fresh organic substrates to gain a competitive advantage, thereby increasing community dominance and reducing evenness. Increased resource inputs may also intensify competition among bacteria for limited niches and readily available carbon sources, suppressing less-adapted taxa. Thus, lower bacterial α diversity does not necessarily indicate ecological functional degradation but may represent community reassembly from a relatively diffuse composition toward taxa with stronger adaptation to available resources. In contrast to bacteria, fungi generally have a greater capacity to degrade complex organic matter, and their filamentous growth enables effective exploitation of spatially heterogeneous organic substrates (Andrews and Harris, 1986; Koskey et al., 2023; Kuzyakov and Razavi, 2019; Maciá-Vicente et al., 2024). Inputs of barley root residues and returned straw increased structural carbon and complex organic substrates, potentially providing more niches for fungi with different degradative capacities and thereby favoring increases in fungal richness and diversity. In addition, changes in rhizosphere pH, nutrient availability, and plant-derived carbon inputs may act as environmental filters affecting colonization and persistence of different fungal taxa. Therefore, the opposite changes observed in bacterial and fungal diversity are more likely the combined result of environmental filtering, resource competition, differences in substrate utilization, and microbial adaptation.

β-diversity analysis based on Bray–Curtis distances further showed significant differentiation in both bacterial and fungal community composition between CK and DM. This indicates that three consecutive years of barley rotation generated a soil microbial community structure distinct from that of the flue-cured tobacco–fallow system. Community reassembly may have been jointly influenced by multiple processes, including selective recruitment of specific microorganisms by barley root exudates, changes in substrate type and resource availability caused by residue inputs, environmental filtering resulting from changes in soil pH and nutrient status, and resource competition and niche differentiation among microorganisms. At the same time, bidirectional feedbacks may occur between plants and microorganisms: the barley rhizosphere environment may select microorganisms adapted to specific carbon sources and nutrient conditions, while enriched microorganisms may in turn further influence organic matter decomposition and nutrient transformation. Because this study did not directly measure root exudate composition, microbial dispersal processes, or the relative contributions of stochastic and deterministic processes to community assembly, these processes should be regarded as ecological interpretations of the observed patterns rather than directly verified mechanisms.

In terms of taxonomic composition, the relative abundances of Pseudomonadota, Acidobacteriota, and some groups within Ascomycota, Basidiomycota, and Mortierellomycota increased under DM. Previous studies have shown that different members of Pseudomonadota can utilize labile carbon sources and participate in multiple nutrient transformation processes, whereas some Acidobacteriota are capable of utilizing complex organic matter and adapting to nutrient-poor environments (Dai et al., 2018; Fierer et al., 2012; Finkel et al., 2017; Lee et al., 2008; Wang et al., 2016). Many members of Ascomycota and Basidiomycota possess strong capacities for degrading plant residues and lignocellulose (Leite et al., 2017; Zhao et al., 2025). Therefore, changes in these taxa are ecologically consistent to some extent with the increased organic carbon input and elevated activities of related enzymes observed in this study. However, phylum-level taxonomic units include many members with highly divergent functions; therefore, specific metabolic functions cannot be inferred solely from changes in phylum-level relative abundance.

At the genus level, *Sphingomonas* and *Burkholderia-Caballeronia-Paraburkholderia* showed relatively high abundance and significant enrichment under DM and were significantly correlated with multiple soil nutrient and enzyme activity variables. Previous studies have reported that some members of these genera possess traits related to organic pollutant degradation, phytohormone production, phosphorus transformation, or other plant growth-promoting functions (Asaf et al., 2020; Bian et al., 2020). The correlation results of the present study indicate strong statistical associations between these two taxa and soil environmental changes generated under barley rotation, supporting their selection as candidate targets for subsequent cultivation, isolation, and functional screening. However, correlation analysis alone cannot demonstrate that these taxa directly regulate soil carbon, nitrogen, phosphorus, or potassium cycling, nor can it demonstrate that they are necessary mediators of the rotation effect. Therefore, they are defined here as “candidate enriched taxa” rather than mechanistically validated “core functional microorganisms”.

Further cultivation and isolation provided strain-level evidence complementary to the community-level associations described above. A total of 255 culturable bacteria were obtained from barley-rotation soil, including six isolates assigned at the 16S rRNA gene level to *Sphingomonas* or *Burkholderia-Caballeronia-Paraburkholderia*. Functional screening under culture conditions showed that these strains exhibited different combinations of phenotypes including growth on nitrogen-free medium, phosphorus transformation, potassium solubilization, zinc solubilization, siderophore production, and organic-substrate degradation. These results demonstrate that the two candidate genera enriched under DM indeed contain culturable members with multiple plant growth-promotion-related phenotypes, providing experimental support for the amplicon community results. However, these culture-based phenotypes only reflect potential functions under specific experimental conditions and do not directly demonstrate that the strains perform the same functions in field soil. In particular, growth on nitrogen-free medium can only serve as a preliminary indication of potential nitrogen-fixing ability and is not equivalent to demonstrating effective biological nitrogen fixation in pot or field environments. The pot reinoculation experiment further showed that inoculation with candidate strains altered flue-cured tobacco growth and nutrient accumulation, with some mixed-inoculation treatments showing relatively pronounced promotion. This provides more direct functional evidence than correlation analysis alone, indicating that these isolates have the potential to influence flue-cured tobacco growth and nutrient uptake under controlled conditions.

This study still has several limitations. Amplicon sequencing cannot directly resolve functional gene expression or metabolic activity; taxonomic identification of the candidate strains still relies mainly on 16S rRNA; key fungi were not functionally validated; and rhizosphere colonization tracking and field reinoculation were not performed. The field performance of microbial inoculants also depends on colonization capacity, environmental adaptability, interactions with indigenous microorganisms, and cross-season stability. Therefore, these isolates should be regarded as candidate microbial resources rather than mature microbial inoculants ready for direct agricultural application. Future studies should integrate whole-genome, metagenomic, or metatranscriptomic approaches with strain-specific quantification and marker-based tracking, and should test ecological functions and the stability of plant growth-promoting effects through multi-location, multi-season field trials.

## 5 Conclusion

This study showed that three consecutive years of flue-cured tobacco–barley rotation were accompanied by increases in soil DOC, AP, AK, and HN, enhanced activities of multiple hydrolytic enzymes, and changes in bacterial and fungal community structure. Sphingomonas and Burkholderia-Caballeronia-Paraburkholderia were selected as candidate enriched bacterial taxa because they were relatively enriched under DM and significantly associated with multiple soil variables. Six culturable isolates obtained from these taxa exhibited different combinations of plant growth-promotion-related phenotypes *in vitro*, and mixed inoculation promoted flue-cured tobacco growth and nutrient accumulation under greenhouse conditions. These results establish a preliminary link between field-scale community changes and functional validation of candidate strains, but they do not yet demonstrate that specific microorganisms directly mediate the improvement of soil properties and flue-cured tobacco growth under barley rotation. Therefore, microbial enrichment should be regarded as one potential mechanism for explaining rotation effects; its actual ecological contribution requires further verification through *in situ* colonization studies, functional omics, and field reinoculation experiments.

## Data Availability

The datasets presented in this study can be found in online repositories. The names of the repository/repositories and accession number(s) can be found in the article/**Supplementary Material**.
