# Supplementary material for: Tobacco–barley rotation reshapes soil microbial communities and enriches culturable bacteria with plant growth-promoting potential
Source: Front Plant Sci. 2026 Oct 28;17:1981771. doi: 10.3389/fpls.2026.1981771 (PMC13751933; doi:10.3389/fpls.2026.1981771)
Supplement: Supplementary file 1 [file DataSheet1.pdf]

Tobacco–barley rotation reshapes soil microbial communities and enriches culturable bacteria with plant growth-promoting potential

Zilin Li[1,†], Chengxiu Fu[2,†], Xun He[1], Zhufeng Shi[2], Yongqin Liao[1], Xufang He[1],Te Pu[2], Yanni Wang[1], Ming Ni[2], You Luo[1], Wei Wang[1], Jing Li[1], Xin He[1],Chenying Li[1], Xuewei Li[1*], Peiwen Yang[2*]

[1]Yunnan Tobacco Company, Lijiang Company, Lijiang, China
[2]Institute of Agricultural Environment and Resources, Yunnan Academy of Agricultural Sciences, Kunming, China
*Corresponding Author: Xuewei Li: ljycjszx@126.com *Peiwen Yang:pwyang2000@126.com
† These authors contributed equally to this work.

Supplementary material

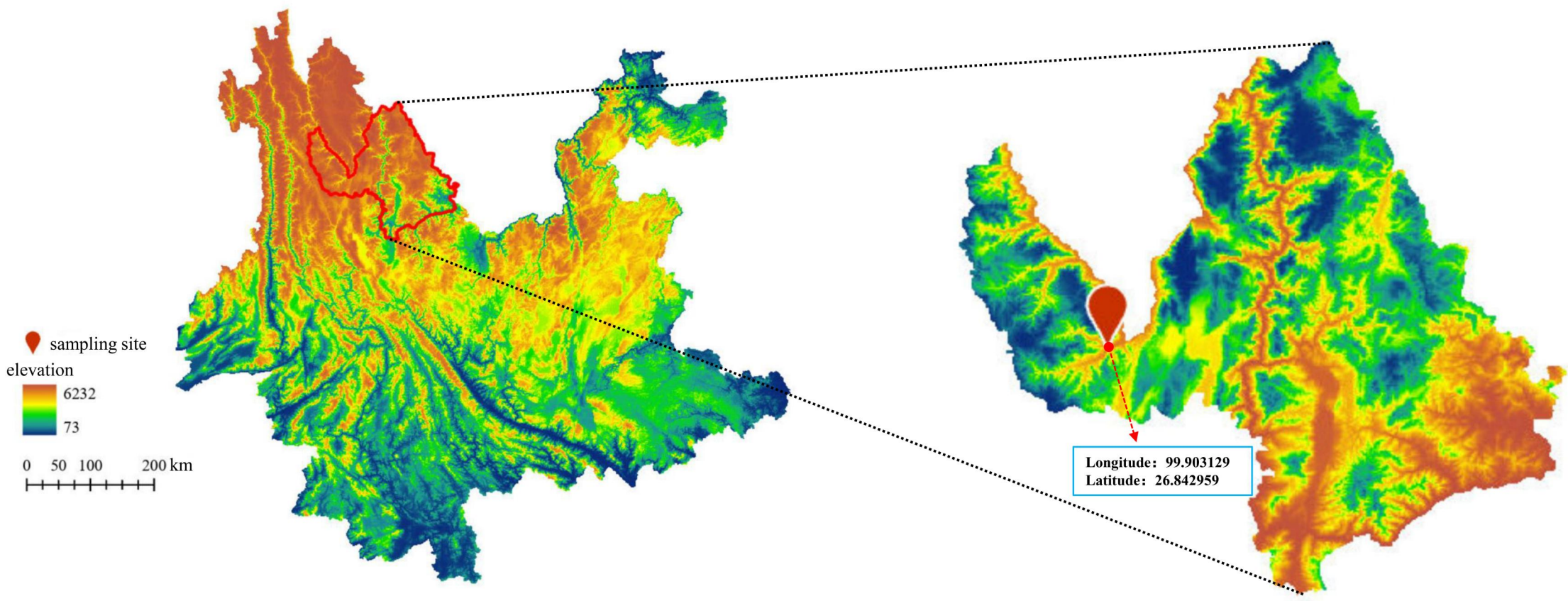

Figure S1. Location of the experimental site

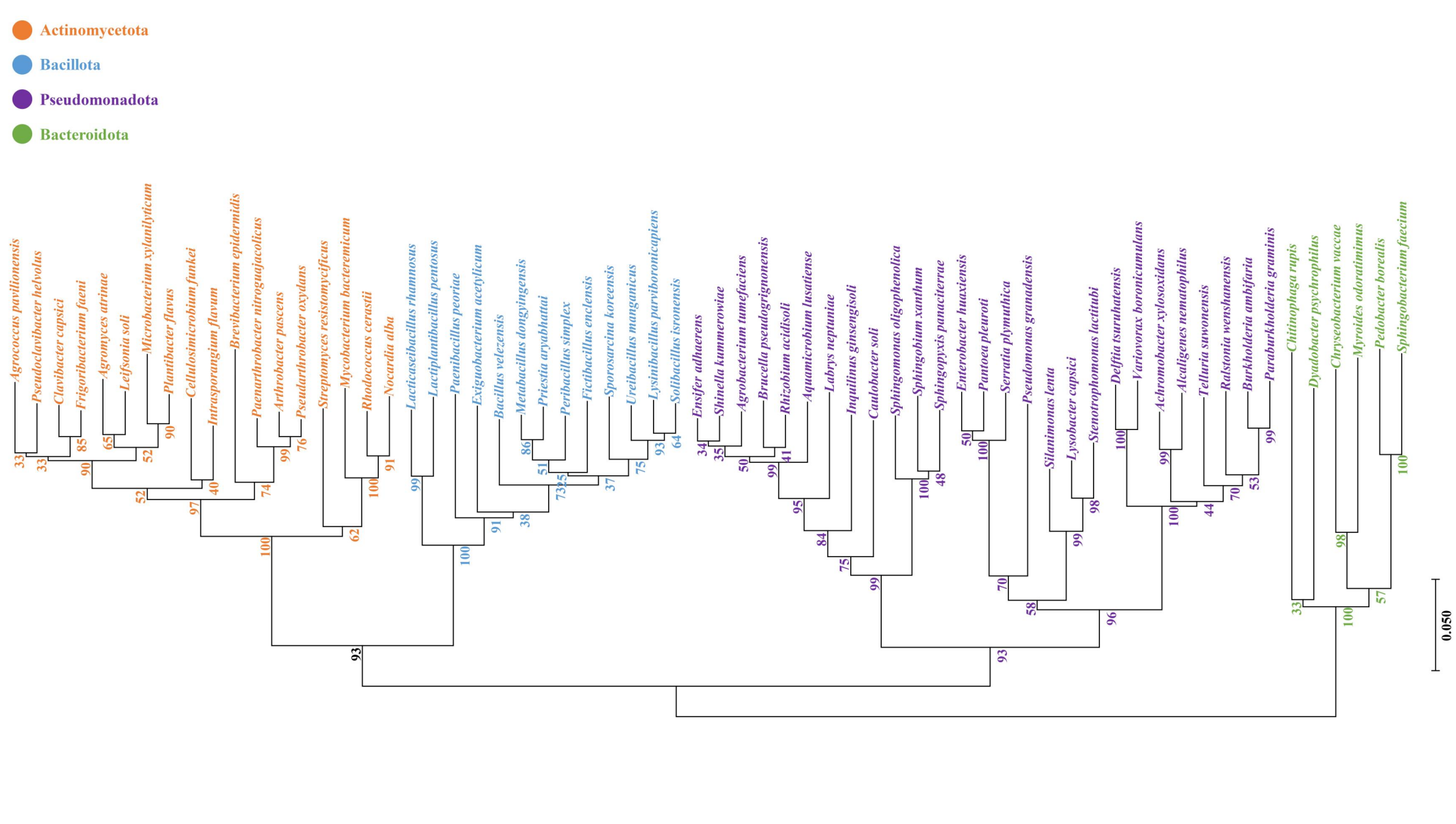

Figure S2. Phylogenetic tree of culturable soil bacteria under flue-cured tobacco-barley rotation system

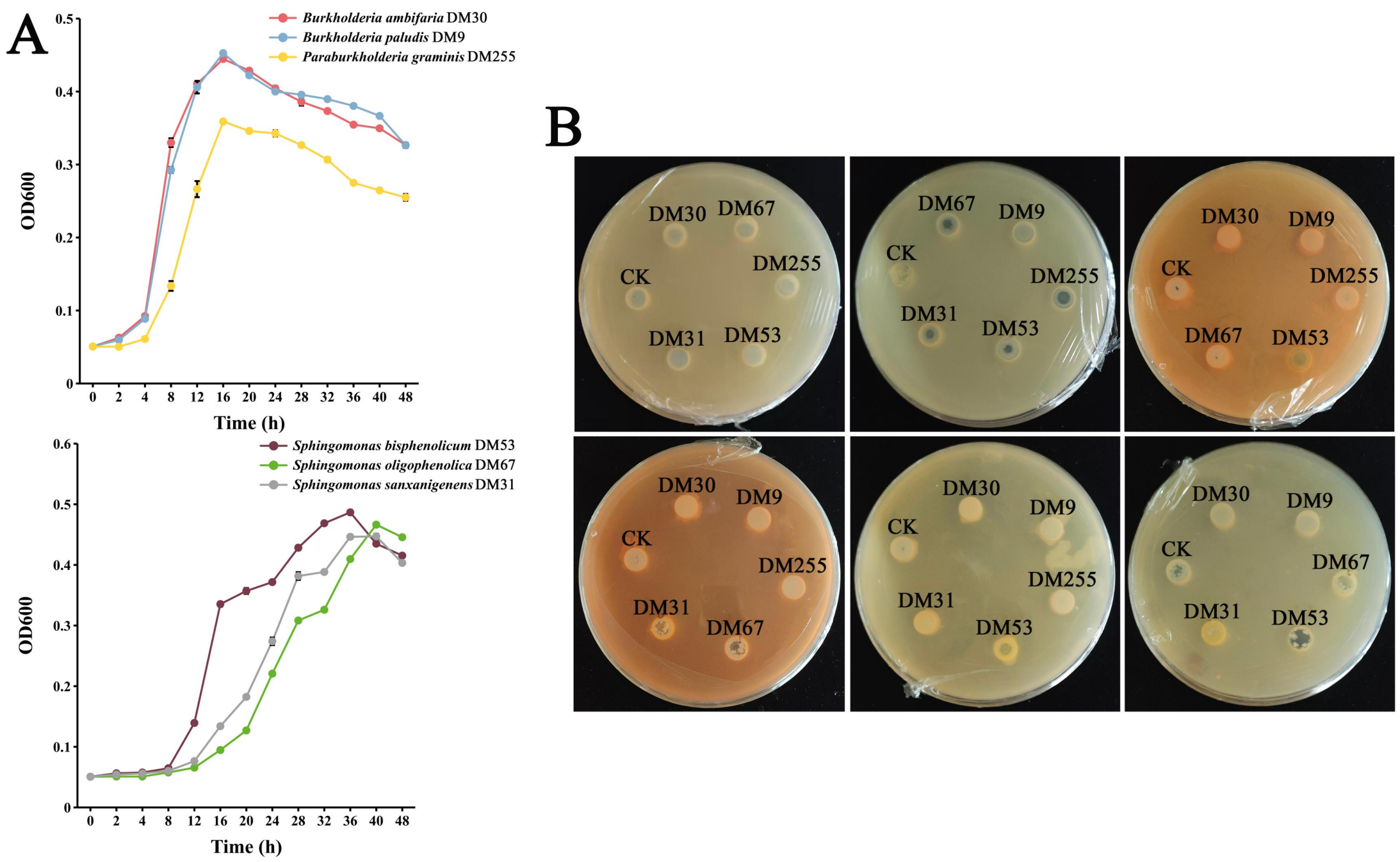

Figure S3. Growth curve of strains (A) and biocompatibility result diagram (B)

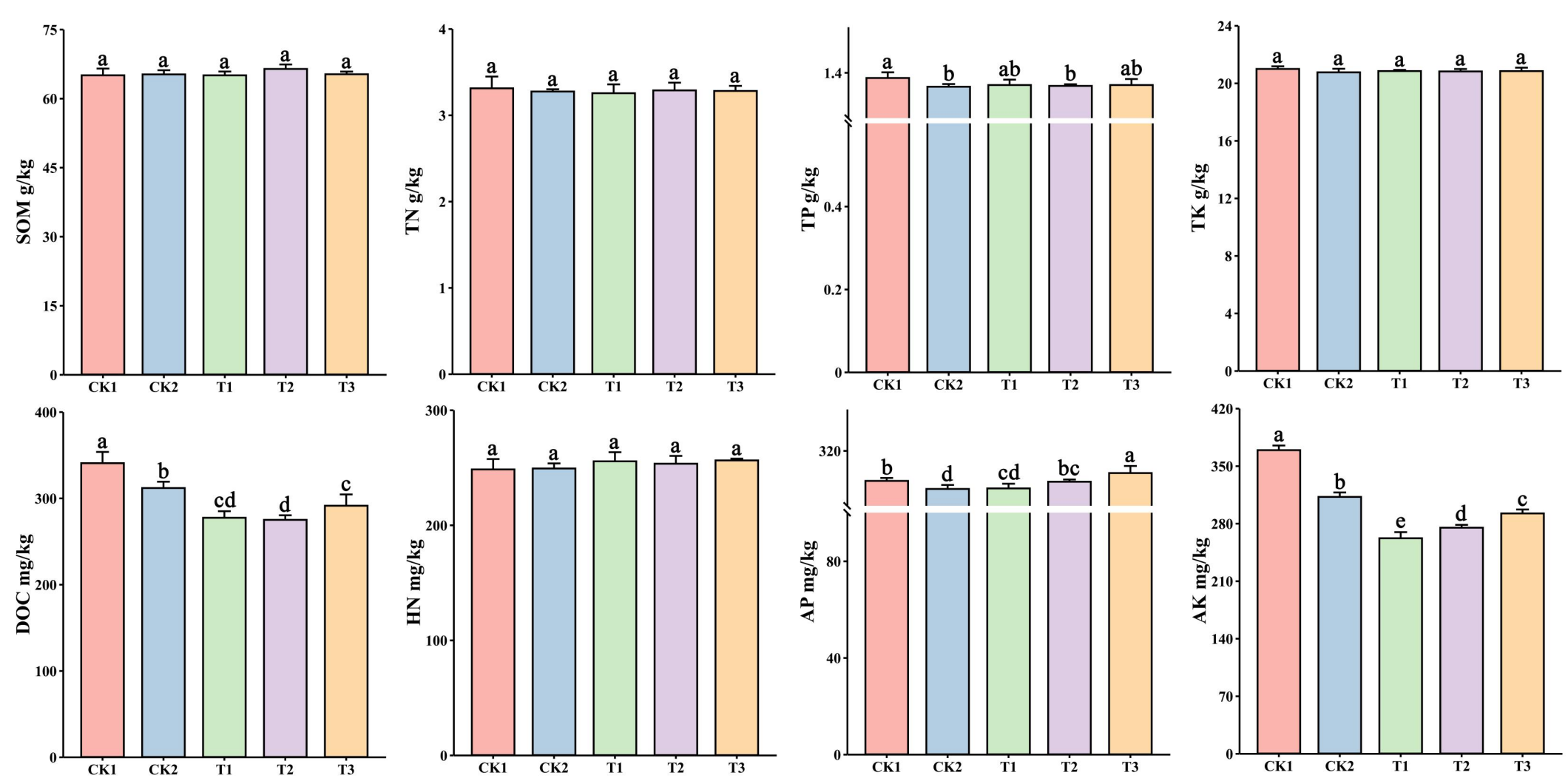

Figure S4. Analysis of soil physicochemical properties after 50 days of treatment with different strains

Note: Each treatment included 10 biological replicates (n = 10). Different letters indicate significant differences ($P < 0.05$, Tukey HSD test). Bars represent means and error bars represent SD.

Table S1. PCR reaction system and conditions

PCR system	PCR conditions

Reagent	Volume	Temperature	Time	Cycle number
DNA template	1 μL	95 ℃	300 s	1
PA（10 μM）	0.5 μL	95 ℃	30 s	
PB（10 μM）	0.5 μL	56 ℃	30 s	35
Green Tap Mix	11 μL	72 ℃	90 s	
ddH_2O	12 μL	72 ℃	600 s	1
Total volume	25 μL	4 ℃		∞

Table S2. Taxonomic status of the six target isolates

ID	Phylum	Top-hit strain	Similarity (%)
DM-9	Pseudomonadota	*Burkholderia paludis* MSh1	99.93
DM-30	Pseudomonadota	*Burkholderia ambifaria* AMMD	99.86
DM-31	Pseudomonadota	*Sphingomonas sanxanigenens* DSM 19645	98.98
DM-53	Pseudomonadota	*Sphingomonas bisphenolicum* AO1	99.49
DM-67	Pseudomonadota	*Sphingomonas oligophenolica* JCM 12082	99.14
DM255	Pseudomonadota	*Paraburkholderia graminis* C4D1M	99.43

Table S3. Functional indices (SI) of the six target isolates

Strain code	plant growth-promoting traits								
	Lignin degradation	Cellulase production	Protease production	Nitrogen fixation	Inorganic phosphorus solubilization	Organic phosphorus mineralization	Potassium solubilization	Zinc solubilization	Siderophore production
DM31	8.14±0.12	1.51±0.06	2.14±0.33	×	×	3.13±0.16	×	×	×
DM53	8.68±0.56	×	3.19±0.34	×	×	1.36±0.11	×	1.87±0.39	3.17±0.26
DM67	8.02±0.32	×	×	×	×	2.87±0.57	×	×	2.83±0.11
DM9	10.13±0.47	2.17±0.13	6.01±0.69	3.07±0.41	1.53±0.21	4.37±0.66	×	5.79±0.68	4.13±0.58
DM30	10.24±0.71	2.25±0.26	2.21±0.18	2.41±0.28	1.81±0.18	5.98±0.37	1.46±0.28	7.02±0.47	4.57±0.63
DM255	10.30±0.88	×	×	4.49±0.45	1.29±0.05	2.95±0.11	3.68±0.31	6.96±0.56	1.69±0.17

Note: Data are presented as mean ± SD (n = 3).×, no functional activity detected.
